# A Systematic Review of Low-Cost Actuator Implementations for Lower-Limb Exoskeletons: a Technical and Financial Perspective

**DOI:** 10.1007/s10846-022-01695-0

**Published:** 2022-08-16

**Authors:** T. Slucock

**Affiliations:** School of Engineering and Digital Arts, University of Kent, Giles Lane, Canterbury, CT2 7NT England

**Keywords:** Actuator, Assistive Devices, Cost, Lower Limb Exoskeleton, Systematic Review, Wearable Robots

## Abstract

A common issue with many commercial rehabilitative exoskeletons and orthoses are that they can be prohibitively expensive for an average individual to afford without additional financial support. Due to this a user may have limited to the usage of such devices within set rehabilitation sessions as opposed to a continual usage. The purpose of this review is therefore to find which actuator implementations would be most suitable for a simplistic, low-cost powered orthoses capable of assisting those with pathologic gait disorders by collating literature from Web of Science, Scopus, and Grey Literature. In this systematic review paper 127 papers were selected from these databases via the PRISMA guidelines, with the financial costs of 25 actuators discovered with 11 distinct actuator groups identified. The review paper will consider a variety of actuator implementations used in existing lower-limb exoskeletons that are specifically designed for the purpose of rehabilitating or aiding those with conditions inhibiting natural movement abilities, such as electric motors, hydraulics, pneumatics, cable-driven actuators, and compliant actuators. Key attributes such as technical simplicity, financial cost, power efficiency, size limitations, accuracy, and reliability are compared for all actuator groups. Statistical findings show that rotary electric motors (which are the most common actuator type within collated literature) and compliant actuators (such as elastic and springs) would be the most suitable actuators for a low-cost implementation. From these results, a possible actuator design will be proposed making use of both rotary electric motors and compliant actuators.

**Supplementary Information:**

The online version contains supplementary material available at 10.1007/s10846-022-01695-0.

## Supplementary Information

Below is the link to the electronic supplementary material.

Supplementary file1 (XLSX 83 KB)
